# Supplementary material for: Low bone mineral density in HIV-positive young Italians and migrants
Source: PLoS One. 2020 Sep 3;15(9):e0237984. doi: 10.1371/journal.pone.0237984 (PMC7470426; doi:10.1371/journal.pone.0237984)

PLOS ONE

Osteoporosis in HIV-positive Young Italians and Migrants

--Manuscript Draft--

**Manuscript Number:**	PONE-D-20-08720
**Article Type:**	Research Article
**Full Title:**	Osteoporosis in HIV-positive Young Italians and Migrants
**Short Title:**	Osteoporosis in HIV
**Corresponding Author:**	Consolato Sergi<br>University of Alberta<br>Edmonton, CANADA
**Keywords:**	HIV; migrant; Osteoporosis; Osteopenia; HCV; Body Mass Density
**Abstract:**	Background: Human immunodeficiency virus (HIV) infected individuals may have osteoporosis. We aimed to evaluate the bone density in naïve antiretroviral (ARV) treated HIV positive patients comparing a native Italian group (ItG) to a Migrants' group (MiG) upon arrival in Italy.<br><br>Methods: We conducted a cross-sectional study on 83 HIV patients with age less than 50 years. Dual-energy X-ray absorptiometry (DXA) within six months from the HIV diagnosis was performed, and the two groups were studied.<br><br>Results: MiG showed osteoporosis more often than ItG (66.7% > 42.4%), especially for the female gender. In ItG, vitamin-D levels were higher than in MiG (14.7 > 11.8 ng/ml). In peripheral blood, low CD4 (<200 cells/μl) was most often detected in MiG.<br><br>Conclusions: HIV positive migrants are at risk of osteoporosis. Both DXA and vitamin-D evaluation should be offered to HIV positive migrants.
**Order of Authors:**	Antonio Cascio
	Claudia Colomba
	Nicola Serra
	Paola Di Carlo
	Giuseppe Lo Re
	Angelo Gambino
	Antonio LoCasto
	Giuseppe Guglielmi
	Nicola Veronese
	Roberto La Galla
	Consolato Sergi
**Additional Information:**	
**Question**	**Response**
**Financial Disclosure**<br><br>Enter a financial disclosure statement that describes the sources of funding for the work included in this submission. Review the submission guidelines for detailed requirements. View published research articles from *PLOS ONE* for specific	The authors received no specific funding for this work.

examples.<br><br>This statement is required for submission and **will appear in the published article** if the submission is accepted. Please make sure it is accurate.<br><br>**Unfunded studies**<br>Enter: *The author(s) received no specific funding for this work.*<br><br>**Funded studies**<br>Enter a statement with the following details:<br>• Initials of the authors who received each award<br>• Grant numbers awarded to each author<br>• The full name of each funder<br>• URL of each funder website<br>• Did the sponsors or funders play any role in the study design, data collection and analysis, decision to publish, or preparation of the manuscript?<br>• **NO** - Include this sentence at the end of your statement: *The funders had no role in study design, data collection and analysis, decision to publish, or preparation of the manuscript.*<br>• **YES** - Specify the role(s) played.<br><br>* typeset	
**Competing Interests**<br><br>Use the instructions below to enter a competing interest statement for this submission. On behalf of all authors, disclose any competing interests that could be perceived to bias this work—acknowledging all financial support and any other relevant financial or non-financial competing interests.<br><br>This statement **will appear in the published article** if the submission is accepted. Please make sure it is accurate. View published research articles from *PLOS ONE* for specific examples.	The authors have declared that no competing interests exist.

Powered by Editorial Manager® and ProduXion Manager® from Aries Systems Corporation

**NO authors have competing interests**<br><br>Enter: *The authors have declared that no competing interests exist.*<br><br>**Authors with competing interests**<br><br>Enter competing interest details beginning with this statement:<br><br>*I have read the journal's policy and the authors of this manuscript have the following competing interests: [insert competing interests here]*<br><br>* typeset	
**Ethics Statement**<br><br>Enter an ethics statement for this submission. This statement is required if the study involved:<br><br>• Human participants<br>• Human specimens or tissue<br>• Vertebrate animals or cephalopods<br>• Vertebrate embryos or tissues<br>• Field research<br><br>Write "N/A" if the submission does not require an ethics statement.<br><br>General guidance is provided below. Consult the submission guidelines for detailed instructions. **Make sure that all information entered here is included in the Methods section of the manuscript.**	The Ethics Committee of the University of Palermo approved the study protocol. Each participant was free to decline or withdraw from the study at any time, and this was explained in their original language or a language that the migrant knew.

Powered by Editorial Manager® and ProduXion Manager® from Aries Systems Corporation

**Format for specific study types**<br><br>**Human Subject Research (involving human participants and/or tissue)**<br>• Give the name of the institutional review board or ethics committee that approved the study<br>• Include the approval number and/or a statement indicating approval of this research<br>• Indicate the form of consent obtained (written/oral) or the reason that consent was not obtained (e.g. the data were analyzed anonymously)<br><br>**Animal Research (involving vertebrate animals, embryos or tissues)**<br>• Provide the name of the Institutional Animal Care and Use Committee (IACUC) or other relevant ethics board that reviewed the study protocol, and indicate whether they approved this research or granted a formal waiver of ethical approval<br>• Include an approval number if one was obtained<br>• If the study involved *non-human primates*, add *additional details* about animal welfare and steps taken to ameliorate suffering<br>• If anesthesia, euthanasia, or any kind of animal sacrifice is part of the study, include briefly which substances and/or methods were applied<br><br>**Field Research**<br><br>Include the following details if this study involves the collection of plant, animal, or other materials from a natural setting:<br>• Field permit number<br>• Name of the institution or relevant body that granted permission	
**Data Availability**<br><br>Authors are required to make all data underlying the findings described fully available, without restriction, and from the time of publication. PLOS allows rare exceptions to address legal and ethical concerns. See the PLOS Data Policy and FAQ for detailed information.	Yes - all data are fully available without restriction

Powered by Editorial Manager® and ProduXion Manager® from Aries Systems Corporation

A Data Availability Statement describing where the data can be found is required at submission. Your answers to this question constitute the Data Availability Statement and **will be published in the article**, if accepted.<br><br>**Important:** Stating 'data available on request from the author' is not sufficient. If your data are only available upon request, select 'No' for the first question and explain your exceptional situation in the text box.<br><br>Do the authors confirm that all data underlying the findings described in their manuscript are fully available without restriction?	
**Describe where the data may be found in full sentences. If you are copying our sample text, replace any instances of XXX with the appropriate details.**<br><br>• If the data are **held or will be held in a public repository**, include URLs, accession numbers or DOIs. If this information will only be available after acceptance, indicate this by ticking the box below. For example: *All XXX files are available from the XXX database (accession number(s) XXX, XXX.*).<br>• If the data are all contained **within the manuscript and/or Supporting Information files**, enter the following: *All relevant data are within the manuscript and its Supporting Information files.*<br>• If neither of these applies but you are able to provide **details of access elsewhere**, with or without limitations, please do so. For example:<br><br>*Data cannot be shared publicly because of [XXX]. Data are available from the XXX Institutional Data Access / Ethics Committee (contact via XXX) for researchers who meet the criteria for access to confidential data.*<br><br>*The data underlying the results presented in the study are available from (include the name of the third party*	Data are available from the University of Palermo Institutional Data Access / Ethics Committee for researchers who meet the criteria for access to confidential data.

and contact information or URL).<br>• This text is appropriate if the data are owned by a third party and authors do not have permission to share the data.<br><br>* typeset	
Additional data availability information:	

Manuscript

Click here to access/download;Manuscript;ASS_OP-Migr_AC_Plos1_TXT-Tabs_20200327.docx

Osteoporosis in HIV-positive Young Italians and Migrants

Antonio Cascio[1], Claudia Colomba[1], Paola Di Carlo[1], Nicola Serra[2], Giuseppe Lo Re[3], Angelo Gambino[3], Antonio Lo Casto[4], Giuseppe Guglielmi[5,6], Nicola Veronese[7], Roberto Lagalla[3], Consolato Sergi [8,9]

1. Department of Health Promotion and Child Health, University Hospital, Palermo, Italy
2. Department of Pediatrics, University Federico II, Naples, Italy
3. Department of Biopathology and Biotechnologies (DiBiMed), University Hospital, 'Paolo Giaccone', Palermo, Italy
4. Department of Biomedicine, Neuroscience and Advanced Diagnostics (BIND), University of Palermo, Italy
5. Department of Radiology, University of Foggia, Viale Luigi Pinto 1, 71100 Foggia, Italy
6. Department of Radiology, Scientific Institute "Casa Sollievo della Sofferenza" Hospital, San Giovanni Rotondo, Foggia, Italy
7. National Research Council, Neuroscience Institute, Aging Branch, Padova, Italy
8. Department of Lab. Medicine and Pathology, University of Alberta, Edmonton, AB, Canada
9. Stollery Children's Hospital, Edmonton, AB, Canada

Antonio Cascio antonio.cascio03@unipa.it
Claudia Colomba claudia.colomba@unipa.it
Nicola Serra nicola.serra@unina.it
Paola Di Carlo paola.dicarlo@unipa.it
Giuseppe Lo Re giuseppe.lore12@gmail.com
Angelo Gambino angelo.gambino01@unipa.it
Antonio Lo Casto antonio.locasto@unipa.it
Giuseppe Guglielmi giuseppe.guglielmi@unifg.it
Nicola Veronese ilmannato@gmail.com
Roberto Lagalla roberto.lagalla@unipa.it
Consolato Sergi sergi@ualberta.ca

Corresponding author: Consolato Sergi, MD, PhD, MPH, FRCPC, FCAP
Department of Laboratory Medicine and Pathology,

University of Alberta, Edmonton, AB, Canada
8440 112 St, NW, Edmonton, AB, Canada
Tel. 1-780-407-7951
Fax. 1-780-407-3009
Email: sergi@ualberta.ca

Disclosure

Preliminary results presented at the HIV Glasgow Conference, Oct. 23-26, 2016, Glasgow, United Kingdom.

Osteoporosis in HIV-positive Young Italians and Migrants

Abstract

Background: Human immunodeficiency virus (HIV) infected individuals may have osteoporosis. We aimed to evaluate the bone density in naïve antiretroviral (ARV) treated HIV positive patients comparing a native Italian group (ItG) to a Migrants' group (MiG) upon arrival in Italy.

Methods: We conducted a cross-sectional study on 83 HIV patients with age less than 50 years. Dual-energy X-ray absorptiometry (DXA) within six months from the HIV diagnosis was performed, and the two groups were studied.

Results: MiG showed osteoporosis more often than ItG (66.7% > 42.4%), especially for the female gender. In ItG, vitamin-D levels were higher than in MiG (14.7 > 11.8 ng/ml). In peripheral blood, low CD4 (<200 cells/μl) was most often detected in MiG.

Conclusions: HIV positive migrants are at risk of osteoporosis. Both DXA and vitamin-D evaluation should be offered to HIV positive migrants.

Abstract Words: 138

Highlights

This study evaluates the frequency of osteoporosis in naïve antiretroviral treatment HIV positive patients and compares the bone density of the native Italian population with that of HIV Migrants upon arrival in Italy. The results show that HIV positive migrants are younger than 50 years and, especially if female, are at risk of osteoporosis.

Keywords: HIV, migrant, Osteoporosis, Osteopenia, HCV, Body Mass Density.

Introduction

Antiretroviral treatment (ARV) has impacted positively on morbidity and mortality among human immunodeficiency virus (HIV)-positive patients. Among HIV-related comorbidities, bone disease has emerged as a significant question in this chronically infected population [1, 2]. The demographic and cultural heterogeneity of the HIV-infected population in terms of race and lifestyles shows how complex is the debate about the prevention and management of the bone disease [2, 3]. Although the number of migrants and refugees crossing the Mediterranean Sea has decreased in 2018 and 2019, this number has been unprecedented in the last ten years compared with the past twenty or thirty years [4-6]. By March 2016, the number of refugees and migrants who had reached Europe by sea was 171,000, and 711 were missing or had lost their lives in the process (UNHCR) [7]. This migratory flow has been raising healthcare concerns for the migrant populations as well as the host countries. Recent epidemiological trend data show that over the last five years' migrants have come from countries with a high rate of HIV, such as sub-Saharan Africa, south, and south-east Asia. They account for a considerable proportion of new HIV diagnoses [8]. Overall, nearly half of all HIV cases are diagnosed late in the European Union/European Economic Area (EU/EEA). In 2015, among cases where information on CD4 cell count was available at the time of diagnosis, 47% were late presenters (CD4 cell count $< 350/mm^3$) when diagnosed. Poor nutritional status and vitamin D deficiency has also been observed in deprived populations [9, 10]. If social deprivation contemplates the reduction of culturally regular interaction between an individual and the rest of society, a network of factors often contributes to his/her social exclusion, including mental illness, poverty, poor education, and low socioeconomic status. The emerging migration flows, mainly from sub-Saharan Africa, have significant consequences for health care in destination countries, both regarding unfamiliar diseases, such as rare infections, and more common chronic non-communicable diseases (NCDs), such as osteoporosis, cardiovascular disorders, diabetes mellitus, and asthma [11].

Osteoporosis is a process characterized by increased bone resorption without reciprocal bone apposition. The decreased bone mass leads to an increased risk of bone fractures, which are particularly as the individuum ages. Currently, lifestyle factors, including alcohol, diet, hormones, physical activity, and smoking, are influencing the bone mass and osteoporosis is affecting younger people than before. The incidence of osteoporosis and osteoporosis-related fractures varies across the EU/EEA, and especially in the Mediterranean area, it is increasing, as it has been evidenced in the literature with a link to nutrition [11, 12].

In consideration of its strategic geographic location between three different continents, Sicily has always been a major Mediterranean crossroads where different peoples and cultures have come together over time. The incidence of bone mineral density loss and related fractures attributed to a specific eating pattern has also been targeted for guidelines and suggestions within the EU/EEA [13]. Here, we report on a cross-sectional study in Sicily equating two age-matched groups, the Italian and a Migrant group. Epidemiologically, this data may reflect a change in the risk of osteoporosis in HIV infected individuals both in today's reality and in the future. Our study aims to emphasize the burden of osteoporosis in naïve ARV HIV positive patients and compare the bone density of the native Italian population group (ItG) with that of HIV Migrants (MiG) upon arrival in Italy.

Materials and Methods

Study design and setting

This investigation is a retrospective cross-sectional study. We gathered data from 83 ARV naïve subjects consecutively admitted between January 2010 and May 2015 at the Sicilian AIDS Center of the University of Palermo who had undergone baseline dual X-ray absorptiometry (DXA) within six months from the HIV. DXA measures bone mineral density (BMD) utilizing two X-ray beams, with different energy levels, which are aimed at the patient's bones (*vide infra*). Of the 83 subjects, 59 were Italian, and 24 were migrant patients living in Italy for less than twelve months. **Table 1** shows the characteristics of enrolled patients. We collected the medical records and entered into an anonymous database as previously reported [13, 14].

CD4+ T-cell count and plasma HIV-RNA levels were assessed as previously reported [14]. The assay of serum 25(OH)D was performed using a Chromo system reagent kit and a chromatographic system equipped with a Waters 1525 Binary HPLC pump connected to a photodiode array detector and detection was carried out at 265 nm. We considered a serum 25(OH)D concentration of 30 μg/L as a threshold value for identifying low levels of vitamin D, as previously reported [15]. The 83 patients, composed by 59.4% males and 40.6% females, with ages into range 30-50, mean 44.2 years old and standard deviation (SD) equal to 4.9 years old, was subdivided into two groups:

- The ItG (Italian group), composed of 59 patients, with ages into range 30-49, mean 43.7 years old and standard deviation (SD) equal to 4.8 years old.
- The MiG (migrant group), composed of 24 patients, with ages into range 32-50, mean 45.4 years old, and standard deviation (SD) equal to 4.8 years old. In detail, 22/24 (92%) migrants came from Africa and 2/24 (8%) from Asia.

All subjects gave their informed consent for inclusion before they started participating in the study, which was conducted by the Declaration of Helsinki. The Ethics Committee of the University of Palermo approved the study protocol. Each participant was free to decline or withdraw from the study at any time, and this was explained in their original language or a language that the migrant knew.

Bone mineral density assessment

BMD was assessed by DXA, using a QDR Discovery Hologic DXA in the femoral neck and DXA in the lumbar spine by total body DXA. For each scan, BMD, T-scores, or Z-scores were recorded as previously reported [14, 16, 17]. DXA measurements were performed in the femur (femoral neck and/or total hip) and lumbar spine in each patient. Since the age of our patients ranged from 30 to 50 years, the T-score was used for all the analyses. The T-score is defined as the difference in the number of standard deviations (SDs) from the mean BMD of a normally distributed, healthy adult reference population [17]. Osteopenia was diagnosed when at least one of the two DXA T-scores was less than −1, and osteoporosis was diagnosed when either femoral neck or lumbar spine, the DXA T-scores were less than −2.5, as recommended by the World Health Organization (WHO) and the National Osteoporosis Foundation [17-20]. In our study, all patients with osteopenia or osteoporosis were considered affected by low bone mineral density.

Statistical analysis

The statistical analysis was performed by Matlab statistical toolbox version 2008 (MathWorks, Natick, MA, USA). Data are presented as number and percentage for categorical variables. Continuous data are expressed as the mean ± standard deviation (SD) unless otherwise specified. The χ^2 test ~~and Fisher's exact test~~ were performed to evaluate significant differences of proportions or percentages between two groups. Fisher's exact test was used where the χ^2 ~~test was not appropriate~~. Instead, to evaluate significant differences of means between ItG and MiG group, we performed with the ~~unpaired~~ t-Student test. Linear correlation analysis was performed, and the test on Pearson's linear correlation coefficient *r* was performed with the t-Student test, ~~under the null hypothesis of Pearson's linear correlation coefficient $r = 0$~~. ~~Finally,~~ all tests with p-value < 0.05 were considered significant

Results

Human Data

Table 1 shows the characteristics of all the patients (ItG + MiG). In **Table 2**, we report the differences between ItG and MiG group and statistical tests. Osteoporosis was significantly more frequent in MiG group in comparison to ItG group (66.7%>42.4%, p-value = 0.046). Particularly, the significant differences were seen in LBD values, (-0.8 > -1.5, p-value =

0.021). Instead, no significant differences were seen in FDB values. Since between MiG and ItG, there was a significant difference for gender (males: 33.3% < 69.5%, p-value = 0.0025), every analysis was performed separately for males and females. Osteoporosis was significantly more frequently found in migrant females than in Italian females (37.5%>1.7%, p-value <0.0001). In examining the HIV infection only, the percentages of HIV patients was significantly greater in Italian males in comparison to migrant males (57.6% > 25%, p-value = 0.007). On the other hand, HIV was most frequent in female migrants in comparison to female Italians (58.3% > 23.7%, p-value < 0.003). For co-infected patients, there were no significant differences in gender between ItG and MiG. Low CD4 values (<200 cells/μl) in peripheral blood were more frequently found in migrant in comparison to Italian patients (44.7% < 83.33%, p-value = 0.0012), while CD4 values into range 200-349 cells/μl were more frequently found in ItG in comparison to MiG patients (50.8% > 16.7%, p-value = 0.0043). There were no significant differences between ItG and MiG concerning high CD4 values (≥ 350 cells/μl). No significant differences in Body Mass Index (BMI) mean values were found between ItG and MiG either. In migrant patients, 25-OH-vitamin D mean values were significantly lower than in Italian patient (14.7 > 11.8 ng/ml, p-value = 0.0466). However, in patients with osteoporosis, the above difference was not significant (15.5 > 11.9 ng/ml, p-value = 0.095). ~~This result near significant level was probably conditioned by a small subgroup of ItG and MiG with osteoporosis described in **table 2**.~~

Table 1. Characteristics of the 83 HIV Study Participants

Parameters	Number
Male/female	49/34
Italian/Migrant	59/24
Age, in years (mean ±SD)	44.2 ±4.9
BMI	
< 18.5	45
18.5 - 25	35
25 - 30	3
CD4 count (cells/µl)	
<200	46
200–349	34
≥ 350	3
AIDS	
Yes	47
Previous fractures	8

HIV=human immunodeficiency; BMI= body mass index; AIDS= acquired immunodeficiency syndrome;

Table 2. Differences between ItG and MiG groups

Parameters	ItG Mean ± SD or percentage	MiG Mean ± SD or percentage	ItG vs. MiG p-value
Nr. Patients	59	24	
Age	43.7±4.8	45.4±4.8	0.15 (T)
Male	43.0±5.1	44.1±4.7	0.37 (T)
Female	45.2±3.6	46.1±4.6	0.34 (T)
Gender (male)	69.5% (41/59)	33.3%(8/24)	0.0025 * (C)
Osteopenia or osteoporosis (OP)	42.4% (25/59)	66.7% (16/24)	0.046 * (C)
Male	40.7% (24/59)	29.2% (7/24)	0.33 (C)
Female	1.7% (1/59)	37.5% (9/24)	<0.0001 * (F)
Co-infected (HIV and HCV)	18.6% (11/59)	16.7% (4/24)	0.83 (C)
Male	11.9% (7/59)	8.3% (2/24)	0.99 (F)
Female	6.8% (4/59)	8.3% (2/24)	0.99 (F)
Mono-infected (HIV)	81.4% (48/59)	83.3% (20/24)	0.83 (C)
Male	57.6% (34/59)	25.0% (6/24)	0.007 * (C)
Female	23.7% (14/59)	58.3% (14/24)	0.003 * (C)
CD4 count (cells/µl)			
<200	44.1% (26/59)	83.33%(20/24)	0.0012 * (C)
200–349	50.8% (30/59)	16.7% (4/24)	0.0043 * (C)
≥ 350	5.1% (3/59)	0.0% (0/24)	0.55 (F)
BMI			
< 18.5	35.6% (21/59)	58.3% (14/24)	0.059 (C)
18.5 – 25 (normal values)	59.3% (35/59)	41.7% (10/24)	0.15 (C)
≥ 25	5.1% (3/59)	0.0% (0/24)	0.55 (F)
Previous fractures	8.5% (5/59)	12.5% (3/24)	0.68 (F)
25-Hydroxy-vitamin D (ng/mL)[a]	14.8±6.4	11.8±5.4	0.0466 * (T)

25-Hydroxyvitamin D (ng/mL)[a] in patients with osteoporosis	15.5±7.1	11.9±5.6	0.095 (T)
Abnormal FBD	18.6% (11/59)	20.8% (5/24)	0.82 (C)
Male	18.6% (11/59)	8.3% (2/24)	0.33 (F)
Female	0.0% (0/59)	12.5% (3/24)	0.033 * (F)
FBD	-0.4±0.8	-0.2±1.0	0.34 (T)
Male	-0.6±0.8	-0.4±0.7	0.29 (T)
Female	0.1±0.6	-0.1±1.1	0.29 (T)
Abnormal LBD	40.0% (23/59)	62.5% (15/24)	0.057 (F)
Male	37.3% (22/59)	29.2% (7/24)	0.48 (C)
Female	1.7% (1/59)	33.3% (8/24)	0.0001 * (F)
LBD	-0.8±1.2	-1.5±1.3	0.021 * (T)
Male	-1.1±1.2	-2.2±1.1	0.0002 * (T)
Female	-0.2±0.9	-1.1±1.3	0.0005 * (T)

* = Significant test; *T =unpaired t- test; C = chi-square test; F* = Fisher's exact test; ItG = Italian group, MiG = migrant group; OP = patients with osteoporosis or osteopenia; FBD = Femoral bone density; LBD = Lumbar bone density; BMI = Body Mass Index

Abnormal FDB values were more frequently observed in migrant females than in Italian women (12.5% > 0.0%, p-value = 0.033); however, the mean values of the T-score was not significantly different between the two groups. For LBD parameter, there was analogous result, i.e., the significant presence of migrant females with abnormal LDB value in comparison to Italian females (33.3% > 1.7%, p-value = 0.0001). In addition, in this case, Italian both females and males had a measure of LBD parameters in mean greater in comparison to migrant females and males respectively (-1.1 > -2.2, p-value = 0.0002; -0.2 > -1.1, p-value = 0.0005). No significant differences between ItG and MiG group were detected for the occurrence of a previous fracture.

In **figure 1** and **figure 2**, the heat maps of LBD and FBD values for ItG and MiG groups are shown, respectively. Every line represents the FBD and LBD values for each patient, and the graduation of the colors are representative of the T-score. Finally, in **figure 3**, we showed the

scatter plots for ItG and MiG group between CD4 values with LBD and FBD (a-d). For every scatters plot, the red points on graphs are individuated by CD4 values and correspondent LBD or FBD T-scores. The blue line is the best linear fit, and the red lines define the 95% prediction interval for the regression curve. For any given value of the independent variable (CD4), this interval represents the 95% probability for the values of the dependent variable (LBD or FBD).

By linear correlation analysis, it results that for ItG group, there was a significant positive linear correlation between CD4 and FBD (r =0.32, p-value = 0.0146) and between CD4 and LBD (r = 0.28, p-value = 0.0319), instead for MiG group there was not a significant correlation between CD4 and FBD (r =0.03, p-value = 0.89) and between CD4 and LBD (r = 0.21, p-value = 0.32). In other words, in Italian patients, an increasing/decreasing of CD4 values implicate an increasing/decreasing of LBD or FBD scores. In contrast, we did not observe these correlations in the MiG group.

Discussion

Our data show that HIV positive migrants (< 1 year) in Italy have osteoporosis more often than HIV positive Italian. We considered LBD and FBD values in both genders and, to the best of our knowledge, this paper is the first DXA investigation to study and assess BMD among the flow of migrants and refugees from countries with a high incidence of HIV infection across the Mediterranean area. In the past, Italian researchers analyzed the prevalence of osteoporosis among migrants who had lived in Milan for at least 15 years, suggesting that there is a high prevalence of osteoporosis and fracture risk in the Southern Italian migrant population relocating in the North of Italy [21]. In this respect, the fact that our study includes only migrants, who were resident in Italy for no more than one year, could offer the opportunity to assess the risk factors, which stress the bone composition in the HIV infected population. A recent review on osteoporosis among HIV positive patients highlighted how an assessment of baseline bone mass loss within the first two years from the start of ARV is often lacking in longitudinal studies and claimed that there is some evidence concerning bone loss within the first year of HIV infection and ART initiation [22, 23].

It has been suggested that female adolescents may harbor more risk factors than other populations, and some factors for bone disorders have been identified in some Islamic countries [24]. Low consumption of dairy products, high consumption of soft drinks, moderate sun exposure, and high prevalence of vitamin D deficiency have been identified among youth as risk factors for early osteoporosis. The nutrition factor is probably intrinsically associated with the geographical region and climate and may aggravate the bone mass loss of HIV-infected youth. Apart from nutrition, HCV co-infection is a risk factor for osteoporosis and fractures in the HIV population. Recently, one of the authors identified a some structural bone defects in HIV/HCV coinfected patients [25], and we reported a high incidence of osteoporosis or osteopenia in HIV mono-infected and HIV/HCV co-infected population than other Italian studies [13, 26, 27].

The findings of a high prevalence of low BMD in infected HIV migrant female and male patients aged less than 50 years of age could suggest that the first screening for osteoporosis should be carried out as soon as possible and before the start of ART. The altered bone remodeling in young HIV positive women may suggest some alertness about our current preventive health care services. Most of the immigrants diagnosed with HIV are pregnant women who have undergone tests for sexually transmitted diseases. Thus, this data calls into question the practice that DXA screening to prevent fracture in HIV and co-infected subjects is carried out at age 50 or older [28]. Regarding the lumbar and femoral T-scores, we found

that low lumbar T-score values were prevalent in HIV/HCV co-infected patients and HIV mono-infected females. Previously, we observed a positive linear correlation between LBD score with pre-fractures in HIV patients [27].

Studies conducted on HIV negative patients have indeed shown how osteophyte formation, bone sclerosis, disk space narrowing, and spondylolisthesis were positively correlated with lumbar spine BMD, while there was no association with femoral neck BMD. Therefore, femoral neck BMD may be more appropriate than lumbar spine BMD for evaluating osteoporosis both in young and senior women [29, 30]. Emerging data show that pregnant and breastfeeding women are associated with early osteoporosis, especially in the thoracolumbar spine [31]. The inclusion of young patients in our study justifies lumbar site determination, and the prevalence of lumbar site osteoporosis T-scores is probably justified by the fact that our sample is younger than in other studies conducted on older populations of HIV negative subjects [23, 32, 33].

As it is known, vitamin D plays a prominent role in bone and mineral metabolism, and it has been suggested to be involved in the pathogenesis of decreased bone mass and fractures in the general population [9]. In HIV patients, several studies have linked vitamin D status with bone disease, low level of BMD [28, 34], as confirmed in our study. Although this study is original and discloses essential steps to improve current management strategies in view of new migrations from underdeveloped countries, there are some limitations. Our study involves a small size of the groups, but the cross-sectional study design still maintains its validity. This study does not permit us to establish a cause-effect relationship from our results or to evaluate the impact of osteoporosis risk factors and/or lifestyle risk factors on the management of bone disease in the HIV population. Thus, a multicentric and prospective study is warranted.

In conclusion, our study raises the question of the need to plan preventive strategies in HIV migrants and refugees, also in the light of the great movement of people from southern Mediterranean areas to northern European countries. The female migrants have to be protected from precocious low BMD with an additional layer of complexity. The approach requires different and specific intervention strategies in the future. Our data confirm that early screening for low BMD in HIV patients is useful [6, 11, 27, 28, 33, 35]. Also, baseline DXA should be performed in HIV migrants and refugees as soon as possible and high-risk groups should be identified, and osteoporosis investigations should be carried out earlier than at 50 years of age.

Legends for Figures

Figure 1. Heat map of FBD and LBD scores for ItG group

Figure 2. Heat map of FBD and LBD scores for MiG group

Figure 3. Scatter plots (a-d) for ItG and MiG group between CD4 values with Lumbar and Femoral Bone densities measured with T-score.

Authors' contributions

AC Conceived and designed the experiments and wrote the manuscript. PdC and CC wrote the manuscript and interpretation of clinical data. AlC and AG performed the initial analysis. GlR and RlG provided DXA data. NV and GG reviewed the first draft. NS reviewed the experimental data, statistical analysis, interpretation of data, and reviewed the last draft of the manuscript. CS reviewed the bone data, and perused the manuscript critically. All authors read and approved the final manuscript.

Conflict of Interests

The authors declare that there is no conflict of interests regarding the publication of this paper.

Acknowledgments

We are deeply grateful to the patients and their families in participating in the study. This study was funded by an intramural grant of the University of Palermo, Italy.

References

1. Mirza FS, Luthra P, Chirch L. Endocrinological aspects of HIV infection. J Endocrinol Invest. 2018;41(8):881-99. doi: 10.1007/s40618-017-0812-x. PubMed PMID: 29313284.
2. Sergi C, Roth SU, Adam S, Otto HF. Mapping a method for systematically reviewing the medical literature: a helpful checklist postmortem protocol of human immunodeficiency virus (HIV)-related pathology in childhood. Pathologica. 2001;93(3):201-7. PubMed PMID: 11433613.
3. Brunner J, Boehler T, Ehemann V, Kassam S, Otto H, Sergi C. Decreased apoptosis despite severe CD4 depletion in the thymus of a human immunodeficiency virus-1 infected child. Klin Padiatr. 2011;223(4):246-8. doi: 10.1055/s-0030-1270514. PubMed PMID: 21271506.
4. Abbas M, Aloudat T, Bartolomei J, Carballo M, Durieux-Paillard S, Gabus L, et al. Migrant and refugee populations: a public health and policy perspective on a continuing global crisis. Antimicrob Resist Infect Control. 2018;7:113. doi: 10.1186/s13756-018-0403-4. PubMed PMID: 30250735; PubMed Central PMCID: PMCPMC6146746.
5. Puchner K, Karamagioli E, Pikouli A, Tsiamis C, Kalogeropoulos A, Kakalou E, et al. Time to Rethink Refugee and Migrant Health in Europe: Moving from Emergency Response to Integrated and Individualized Health Care Provision for Migrants and Refugees. Int J Environ Res Public Health. 2018;15(6). doi: 10.3390/ijerph15061100. PubMed PMID: 29843445; PubMed Central PMCID: PMCPMC6024938.
6. Schilling T, Rauscher S, Menzel C, Reichenauer S, Muller-Schilling M, Schmid S, et al. Migrants and Refugees in Europe: Challenges, Experiences and Contributions. Visc Med. 2017;33(4):295-300. doi: 10.1159/000478763. PubMed PMID: 29034258; PubMed Central PMCID: PMCPMC5624244.
7. WHO. Stepping up action on refugee and migrant health. Towards a WHO European framework for collaborative action. Rome, Italy: World Health Organization, 2015 23-24 November 2015. Report No.
8. Camoni L, Raimondo M, Dorrucci M, Regine V, Salfa MC, Suligoi B, et al. Estimating minimum adult HIV prevalence: a cross-sectional study to assess the characteristics of people living with HIV in Italy. AIDS Res Hum Retroviruses. 2015;31(3):282-7. doi: 10.1089/AID.2014.0154. PubMed PMID: 25432098; PubMed Central PMCID: PMCPMC4348082.
9. Zhang M, Shen F, Petryk A, Tang J, Chen X, Sergi C. "English Disease": Historical Notes on Rickets, the Bone-Lung Link and Child Neglect Issues. Nutrients. 2016;8(11). doi: 10.3390/nu8110722. PubMed PMID: 27854286; PubMed Central PMCID: PMCPMC5133108.
10. Sergi C, Linderkamp O. Pathological case of the month: classic rickets in a setting of significant psychosocial deprivation. Arch Pediatr Adolesc Med. 2001;155(8):967-8. PubMed PMID: 11483129.
11. WHO. Strategy and action plan for refugee and migrant health in the WHO European Region. Copenhagen, Denmark: World Health Organization, 2016 12–15 September 2016. Report No.
12. Messina OD, Vidal Wilman M, Vidal Neira LF. Nutrition, osteoarthritis and cartilage metabolism. Aging Clin Exp Res. 2019. doi: 10.1007/s40520-019-01191-w. PubMed PMID: 30982220.
13. Li Vecchi V, Soresi M, Giannitrapani L, Mazzola G, Colletti P, Amico ID, et al. Associated Factors and Liver Disease Severity for Decreased Bone Mineral Density in HIV Mono- and HIV/HCV Co-infected Patients. J AIDS Clin Res. 2015;6:438. doi: 10.4172/2155-6113.1000438.

14. Li Vecchi V, Soresi M, Giannitrapani L, Mazzola G, La Sala S, Tramuto F, et al. Dairy calcium intake and lifestyle risk factors for bone loss in hiv-infected and uninfected Mediterranean subjects. BMC Infect Dis. 2012;12:192. doi: 10.1186/1471-2334-12-192. PubMed PMID: 22894751; PubMed Central PMCID: PMCPMC3447655.
15. Petta S, Ferraro D, Camma C, Cabibi D, Di Cristina A, Di Marco V, et al. Vitamin D levels and IL28B polymorphisms are related to rapid virological response to standard of care in genotype 1 chronic hepatitis C. Antivir Ther. 2012;17(5):823-31. doi: 10.3851/IMP2100. PubMed PMID: 22505587.
16. Diacinti D, Guglielmi G. Vertebral morphometry. Radiol Clin North Am. 2010;48(3):561-75. doi: 10.1016/j.rcl.2010.02.018. PubMed PMID: 20609892.
17. WHO. Assessment of fracture risk and its application to screening for postmenopausal osteoporosis: report of a WHO study group [meeting held in Rome from 22 to 25 June 1992]. Geneva, Switzerland: World Health Organization, 1994 22 to 25 June 1992. Report No.
18. Denova-Gutierrez E, Mendez-Sanchez L, Munoz-Aguirre P, Tucker KL, Clark P. Dietary Patterns, Bone Mineral Density, and Risk of Fractures: A Systematic Review and Meta-Analysis. Nutrients. 2018;10(12). doi: 10.3390/nu10121922. PubMed PMID: 30563066; PubMed Central PMCID: PMCPMC6316557.
19. Bazzocchi A, Ponti F, Albisinni U, Battista G, Guglielmi G. DXA: Technical aspects and application. Eur J Radiol. 2016;85(8):1481-92. doi: 10.1016/j.ejrad.2016.04.004. PubMed PMID: 27157852.
20. Cosman F, de Beur SJ, LeBoff MS, Lewiecki EM, Tanner B, Randall S, et al. Clinician's Guide to Prevention and Treatment of Osteoporosis. Osteoporos Int. 2014;25(10):2359-81. doi: 10.1007/s00198-014-2794-2. PubMed PMID: 25182228; PubMed Central PMCID: PMCPMC4176573.
21. Varenna M, Binelli L, Zucchi F, Rossi V, Sinigaglia L. Prevalence of osteoporosis and fractures in a migrant population from southern to northern Italy: a cross-sectional, comparative study. Osteoporos Int. 2003;14(9):734-40. doi: 10.1007/s00198-003-1438-8. PubMed PMID: 12856113.
22. Goh SSL, Lai PSM, Tan ATB, Ponnampalavanar S. Reduced bone mineral density in human immunodeficiency virus-infected individuals: a meta-analysis of its prevalence and risk factors: supplementary presentation. Osteoporos Int. 2018;29(7):1683. doi: 10.1007/s00198-018-4379-y. PubMed PMID: 29737369.
23. Goh SSL, Lai PSM, Tan ATB, Ponnampalavanar S. Reduced bone mineral density in human immunodeficiency virus-infected individuals: a meta-analysis of its prevalence and risk factors. Osteoporos Int. 2018;29(3):595-613. doi: 10.1007/s00198-017-4305-8. PubMed PMID: 29159533.
24. Al-Raddadi R, Bahijri S, Borai A, AlRaddadi Z. Prevalence of lifestyle practices that might affect bone health in relation to vitamin D status among female Saudi adolescents. Nutrition. 2018;45:108-13. doi: 10.1016/j.nut.2017.07.015. PubMed PMID: 29129230.
25. Lo Re V, 3rd, Lynn K, Stumm ER, Long J, Nezamzadeh MS, Baker JF, et al. Structural Bone Deficits in HIV/HCV-Coinfected, HCV-Monoinfected, and HIV-Monoinfected Women. J Infect Dis. 2015;212(6):924-33. doi: 10.1093/infdis/jiv147. PubMed PMID: 25754980; PubMed Central PMCID: PMCPMC4548465.
26. Di Carlo P, Siracusa L, Mazzola G, Colletti P, Soresi M, Giannitrapani L, et al. Vitamin D and Osteoporosis in HIV/HCV Coinfected Patients: A Literature Review. Int J Endocrinol. 2015;2015:969040. doi: 10.1155/2015/969040. PubMed PMID: 26273302; PubMed Central PMCID: PMCPMC4530270.
27. Serra N, Li Vecchi V, Soresi M, Giannitrapani L, Montalto G, Lo Casto A, et al. Osteoporosis risk factors in HIV positive women with osteoporosis: a retrospective analysis. Pharmacology Online. 2017;1:51-61.

28. IOM. Global Migration Indicators 2018. Berlin, Germany: International Organization for Migration, 2018 27 May 2016. Report No.
29. Davidson N, Sowden D. Evaluation of screening practices for low bone mass and prevalence of osteoporosis and fractures in people living with HIV attending a sexual health clinic. Intern Med J. 2019. doi: 10.1111/imj.14357. PubMed PMID: 31081166.
30. Sergi C, Shen F, Liu SM. Insulin/IGF-1R, SIRT1, and FOXOs Pathways-An Intriguing Interaction Platform for Bone and Osteosarcoma. Front Endocrinol (Lausanne). 2019;10:93. doi: 10.3389/fendo.2019.00093. PubMed PMID: 30881341; PubMed Central PMCID: PMCPMC6405434.
31. Wimalawansa SJ, Razzaque MS, Al-Daghri NM. Calcium and vitamin D in human health: Hype or real? J Steroid Biochem Mol Biol. 2018;180:4-14. doi: 10.1016/j.jsbmb.2017.12.009. PubMed PMID: 29258769.
32. Prinapori R, Rosso R, Di Biagio A, Nicolini L, Giacobbe DR, De Hoffer L, et al. Phalangeal quantitative ultrasound: cheaper methods for screening and follow-up of bone pathologies in HIV-infected women? New Microbiol. 2013;36(4):345-51. PubMed PMID: 24177296.
33. Greig JM, Anderson J. Optimizing antiretroviral therapy for women living with HIV. Curr Opin Infect Dis. 2014;27(1):46-52. doi: 10.1097/QCO.0000000000000033. PubMed PMID: 24322593.
34. Atteritano M, Mirarchi L, Venanzi-Rullo E, Santoro D, Iaria C, Catalano A, et al. Vitamin D Status and the Relationship with Bone Fragility Fractures in HIV-Infected Patients: A Case Control Study. Int J Mol Sci. 2018;19(1). doi: 10.3390/ijms19010119. PubMed PMID: 29301284; PubMed Central PMCID: PMCPMC5796068.
35. Kyvernitakis I, Reuter TC, Hellmeyer L, Hars O, Hadji P. Subsequent fracture risk of women with pregnancy and lactation-associated osteoporosis after a median of 6 years of follow-up. Osteoporos Int. 2018;29(1):135-42. doi: 10.1007/s00198-017-4239-1. PubMed PMID: 28965212.

Figure 1

Click here to access/download;Figure;ASS_OP-Migr_AC_Plos1_Fig1_20200326 (ItG).tif

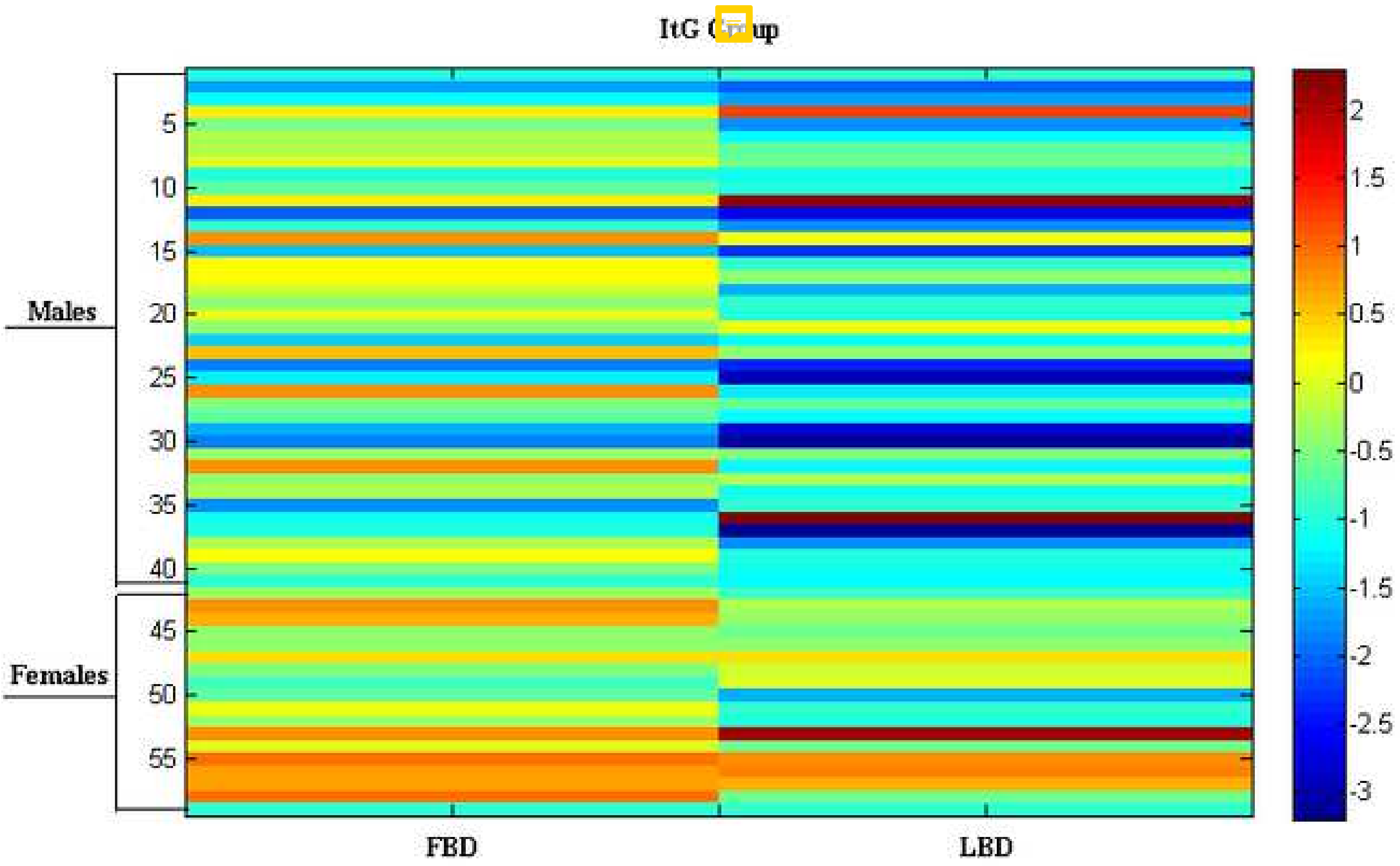

Figure 2

Click here to access/download;Figure;ASS_OP-Migr_AC_Plos1_Fig2_20200326 (MiG).tif

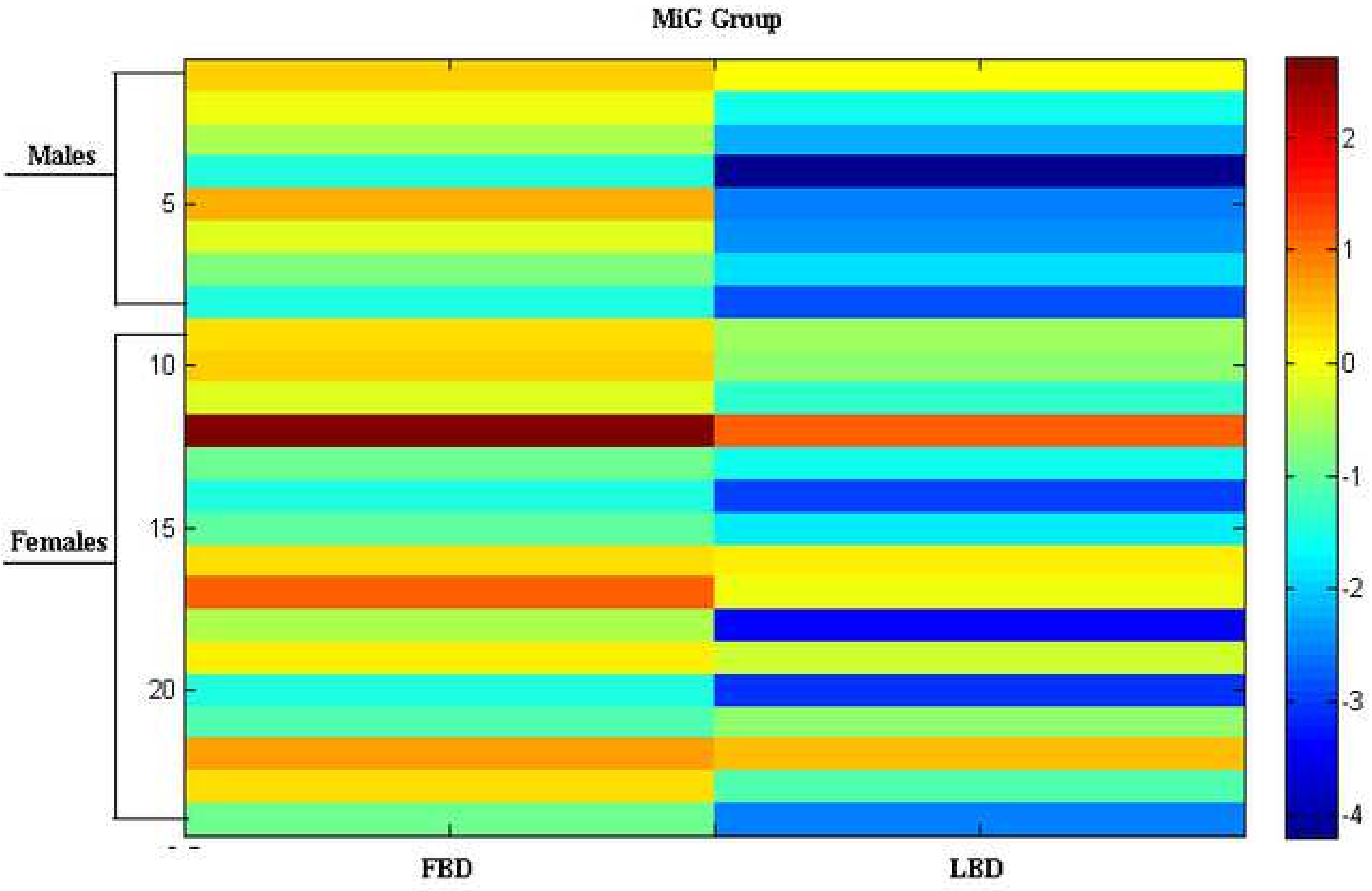

Figure 3

Click here to access/download;Figure;ASS_OP-Migr_AC_Plos1_Fig3_20200326.tif

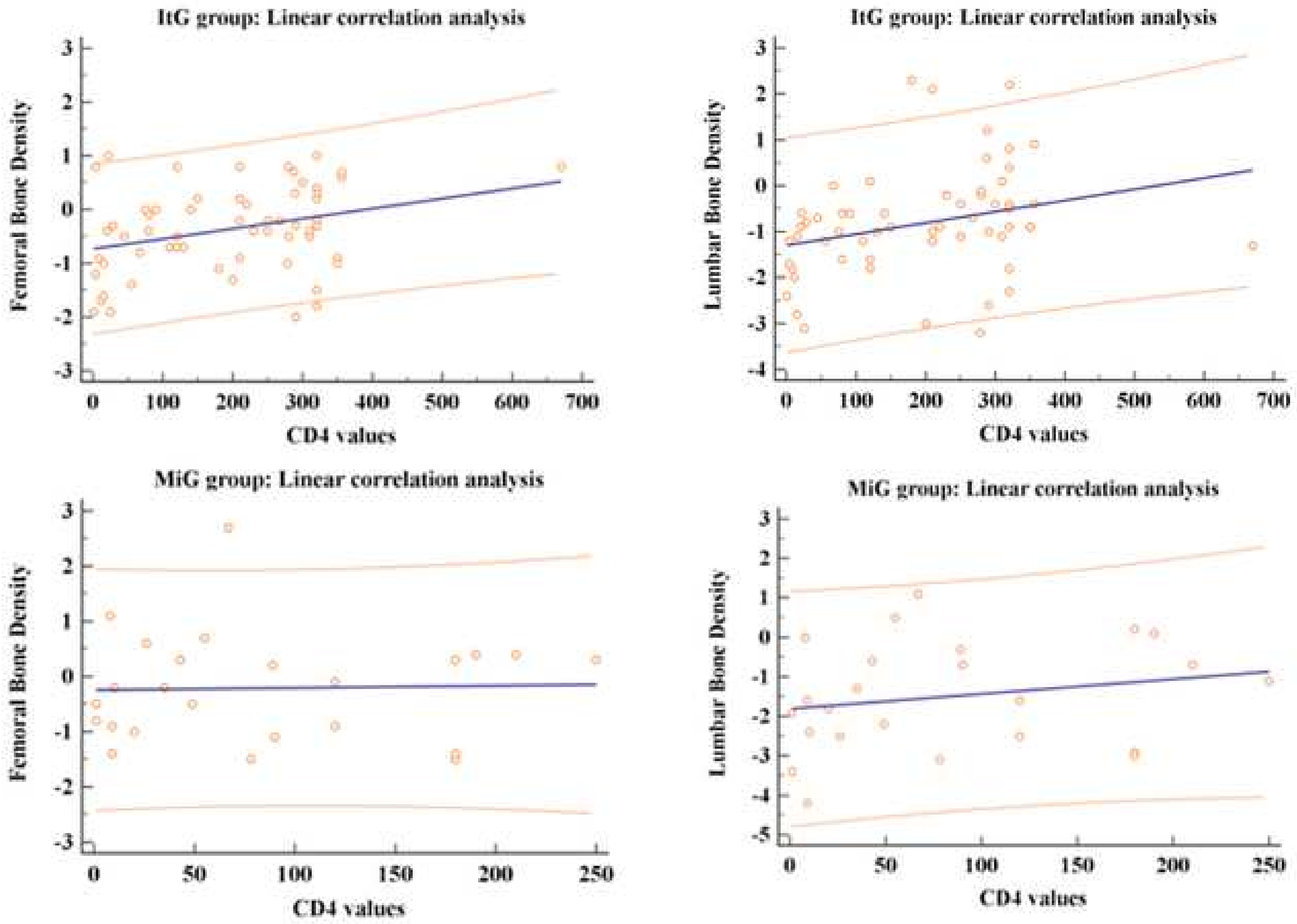

Supplement: Attachment — Submitted filename: PONE-D-20-08720_HD.pdf [file pone.0237984.s001.pdf]
