# Supplementary material for: Low bone mineral density in HIV-positive young Italians and migrants
Source: PLoS One. 2020 Sep 3;15(9):e0237984. doi: 10.1371/journal.pone.0237984 (PMC7470426; doi:10.1371/journal.pone.0237984)
Supplement: Attachment — Submitted filename: PONE-D-20-08720_comments.docx [file pone.0237984.s002.docx]

**PONE-D-20-08720
"Osteoporosis in HIV-positive Young Italians and Migrants"**

**Recommendation:** reject

**General Comments:**

Much better suited (after major revision) for an HIV-specific journal – as there is little to be learned for a general medical audience from this study. Would rewrite this with the help of a native English speaker into a much more descriptive paper – not “hunting” for statistical significance.

**Specific Comments:**

(Relatively) small comparative cross-sectional study on bone mineral density in HIV-infected, ART-naïve (mostly African) recent migrants and native Italians from Sicily.

The major problems with this study are:

- no underlying hypothesis

- no clear inclusion/exclusion criteria (who got a DXA scan and why?) what was the exact age range in each group?

- the major “finding” was a much higher rate of osteoporosis/osteopenia in migrant women compared with Italian women – that seemed to be explained by low rate of osteopenia in Italian women in their sample and likely also by much more advanced HIV disease in migrant women – but the authors unfortunately did not delineate HIV-specific and other osteoporosis by gender *and* migrant status. Important questions: menopause status? History of TB?

Minor points: see comments in edited .pdf file.

- Would elaborate on implications for ART management and longitudinal monitoring in discussion and discuss osteoporosis treatment
