# Supplementary material for: Low bone mineral density in HIV-positive young Italians and migrants
Source: PLoS One. 2020 Sep 3;15(9):e0237984. doi: 10.1371/journal.pone.0237984 (PMC7470426; doi:10.1371/journal.pone.0237984)
Supplement: Attachment — Submitted filename: Reviewers Response_R2.rtf [file pone.0237984.s004.rtf]

Reviewer #2: General points: You went through great lengths to address most of the reviewers' points- thanks!
Still, PLEASE find somebody who is proficient in English or use the F7 key in Microsoft Word to correct the many glaring grammatical mistakes in your paper. THANKS!
Thank you for your comments. Yes, we revised the manuscript carefully, and one colleague with English as a native language went through it.

[Reviewer 2]: I stick with my opinion that your main finding, the difference in BMD between migrants and native Italians, concerns only women, and it may or may not have anything to do with HIV. If no data menopause status were available for your study, you should state so in Methods and mention this as a limitation in your Discussion. An alternative explanation would be differences in sun exposure, and this could involve cultural factors, depending on the origin/culture/religious habits of the migrant women in your study. 
Thank you for your comment, and we changed the manuscript appropriately. According to the menopause definition (amenorrhea for ≥12 consecutive months with symptoms suggestive of menopause and in which other causes of amenorrhea have been ruled out and/or the FSH level is elevated) our female sample showed menopause status in 11/16 (68.75%) of female migrant and 12/18 of Italian female (66%). We added this data to the manuscript.
We changed the Discussion as well.
As reported in other studies, we observed early natural menopause status both in Italian than in migrant women. This finding was analogous to both groups. Apart from HIV-related immunologic status (CD4 count and viral load) other socio-demographic variables (marital status, parity, education, or income) and religious clothing of the female migrant sample may have influenced the low BMD observed in our study. 
(Guilherme Amaral Calvet, Beatriz Gilda Jegerhorn Grinsztejn, Marcel de Souza Borges Quintana, Monica Derrico, Emilia Moreira Jalil, Andrea Cytryn, Angela Cristina Vasconcelos de Andrade, Ronaldo Ismerio Moreira, Marcelo Ribeiro Alves, Valdiléa Gonçalves Veloso dos Santos, Ruth Khalili Friedman, Predictors of early menopause in HIV-infected women: a prospective cohort study, American Journal of Obstetrics and Gynecology, Volume 212, Issue 6, 2015, Pages 765.e1-765.e13, ISSN 0002-9378, https://doi.org/10.1016/j.ajog.2014.12.040. (http://www.sciencedirect.com/science/article/pii/S0002937814024995)

[Reviewer 2]: Table 2 with the 25-OH Vitamin D values is confusing me, particularly the "abnormal" (<12 ng/mL?) category doesn't seem to make sense - was really everybody abnormal??? Migrant women seem to have much higher levels than men, which is unexpected - the opposite was true for native Italians. PLEASE, look at your source data again and make sure that you are publishing correct numbers....
ITA MIG
Abnormal 25-OH-vitD 100% (59/59) 100% (24/24) 1.00 (F)
Male 69.5% (41/59) 33% (8/24) 0.0024* (C)
Female 31.5% (18/59) 66.7% (16/24) 0.0024* (C)
25-OH-vitD (ng/mL) 14.8±6.4 11.8±5.4 0.0466 * (T)
Male 15.6±6.9 9.8±5.9 0.0324 * (T)
Female 12.9±4.4 12.8±5.2 0.94 (T)
Thank you, we reviewed all data again and made the corrections. All data have been verified and validated using a triple code identifier. All data are also available for consultation. 
Concerning the normal range of Vitamin D, it was corrected in [30-80] (ng/mL) with an analogous concentration rate in the paper. About the statistical tests, all patients in MiG and ItG had a low value of vitamin D, but in MiG was significantly lower than the ItG (T-test). Since the results were incomplete, we adopted a comparison of proportions. We tested the proportion rates between MiG and ItG for males and females:

Male (with abnormal value)      69.5% (41/59)  vs.      33% (8/24)      0.0024* (C)
Female (with abnormal value)    31.5% (18/59)  vs.      66.7% (16/24)   0.0024* (C)
-------------------------------------------------------------------------------------------------------------
                               ItG 100% (59/59)  vs. MiG 100% (24/24)
We consulted our statisticians to answer to your question.

[Reviewer 2]: Finally, I like the color-coded z-score Figures that are juxtaposing the ITA and the MIG group. However, I think they ought to be c o m p l e t e l y rearranged - make one figure for femoral and one for lumbar BMD, ITA group left, MIG group right. And then SORT the individual values for each group (maintaining stratification by gender) from lowest to highest. This will make the most meaningful graphical depiction of your findings.
Thank you for your suggestion. We agree that such figures would make the best of our data, but to obtain this type of figure, there must be groups of the same size, according to our statisticians. To represent a heatmap, a 2-column matrix with the same number of rows was used. Finally, we plot the figure in this way, but the gradualness of the colors in some parts was difficult to appreciate. Therefore, we decided to consider the order of the patients, as reported in the database. We consulted our statisticians to answer to your question and reviewed the manuscript with them.

[Reviewer 2]: For the CD4 / BMD scatter plots, I would keep the scale of the x-axis for both groups identical, i.e. considerably shorten the x-axis for the MIG group. This will make the groups easier to compare.
Thank you for your suggestion. In this case, we did consider for all scatterplots an analogous scale of the x-axis. Indeed, the scale of values on the x-axes, makes the differences evident between MiG and ItG. We consulted our statisticians to answer to your question and reviewed the manuscript with them.
